# Scalable purification of extracellular vesicles with high yield and purity using multimodal flowthrough chromatography

**DOI:** 10.1002/jex2.138

**Published:** 2024-01-31

**Authors:** Scott E. Bonner, Simonides I. van de Wakker, William Phillips, Eduard Willms, Joost P. G. Sluijter, Andrew F. Hill, Matthew J. A. Wood, Pieter Vader

**Affiliations:** ^1^ Department of Paediatrics University of Oxford Oxford UK; ^2^ Department of Experimental Cardiology University Medical Center Utrecht, Utrecht University Utrecht The Netherlands; ^3^ Department of Biochemistry and Chemistry La Trobe Institute for Molecular Science La Trobe University Bundoora Victoria Australia; ^4^ Institute for Health and Sport Victoria University Melbourne Victoria Australia; ^5^ CDL Research University Medical Center Utrecht Utrecht The Netherlands

**Keywords:** extracellular vesicles, exosomes, multimodal flowthrough chromatography, purification, size exclusion chromatography

## Abstract

Extracellular vesicles (EVs) are cell derived membranous nanoparticles. EVs are important mediators of cell–cell communication via the transfer of bioactive content and as such they are being investigated for disease diagnostics as biomarkers and for potential therapeutic cargo delivery to recipient cells. However, existing methods for isolating EVs from biological samples suffer from challenges related to co‐isolation of unwanted materials such as proteins, nucleic acids, and lipoproteins. In the pursuit of improved EV isolation techniques, we introduce multimodal flowthrough chromatography (MFC) as a scalable alternative to size exclusion chromatography (SEC). The use of MFC offers significant advantages for purifying EVs, resulting in enhanced yields and increased purity with respect to protein and nucleic acid co‐isolates from conditioned 3D cell culture media. Compared to SEC, significantly higher EV yields with similar purity and preserved functionality were also obtained with MFC in 2D cell cultures. Additionally, MFC yielded EVs from serum with comparable purity to SEC and similar apolipoprotein B content. Overall, MFC presents an advancement in EV purification yielding EVs with high recovery, purity, and functionality, and offers an accessible improvement to researchers currently employing SEC.

## 1 INTRODUCTION

Extracellular vesicles (EVs) are membrane bound nanoparticles released by all cell types. EVs assume a wide range of sizes and are often divided into different categories based on their biogenesis (Van Niel et al., 2018). Of note, based on biogenesis the two most handled categories of EVs are ectosomes and exosomes. Ectosomes are formed by the outward budding of cell membranes (Tricarico et al., 2017), while exosomes are formed via the endo‐lysosomal pathway and are released from cells when multivesicular bodies fuse with the cell membrane (Harding et al., 1984; Pan et al., 1985). When first discovered, EVs were regarded as a mechanism for eliminating waste from cells (Johnstone et al., 1987). However, following studies in the 1990s first documenting physiological activity of EVs (Raposo et al., 1996), EVs have since been evidenced as important mediators of intercellular communication in health and disease through the transfer of their protein, lipid and nucleic acid cargoes to recipient cells (Cicero et al., 2015; Colombo et al., 2014; Lee et al., 2012; Witwer et al., 2013; Yáñez‐Mó et al., 2015). In addition to the ability of EVs to transfer their bioactive cargoes, EVs are biocompatible and have been shown to cross biological barriers (Elsharkasy et al., 2020). Importantly, EVs can be engineered for therapeutic use and drug delivery (Murphy et al., 2019).

Historically, EVs have been purified from cell culture supernatant and biofluids using a variety of different techniques including various precipitation, ultracentrifugation, affinity and chromatographic techniques, each with their own applications, advantages and disadvantages (Sidhom et al., 2020; Zhou et al., 2020). The most common method of EV purification in the last few decades has been ultracentrifugation (Gardiner et al., 2016; Royo et al., 2020). However, as new techniques have been developed to characterise EVs and the understanding of EV characteristics has improved, ultracentrifugation has been identified as yielding EV preps that can obtain co‐isolated proteins, lipoproteins and lipoprotein particles, and can damage and malform EVs which impairs their functionality (Baranyai et al., 2015; Mol et al., 2017). This has coincided with a decrease in the use of ultracentrifugation in favour of other techniques, including a significant increase in the use of size exclusion chromatography (SEC) which became the second most commonly used isolation technique among EV researchers worldwide in 2020 (Royo et al., 2020). SEC employs columns packed with inert, porous, resin beads that can be perfused with a liquid sample to separate its components based on size. Components larger than the resin pores flow around the beads and elute earlier. Smaller components can enter the beads and have a longer path though the column and are eluted later. Components can be separated to varying degrees depending on the resolving capabilities of the resin. For conventional EV purification from 2D culture SEC generates clean EV preps that are intact and functional (Böing et al., 2014; Hong et al., 2012; Lindqvist & Storgårds, 1955). However, as the need to upscale EV production using bioreactors arises, such as for attaining clinically relevant concentrations of EVs, or the need to purify EVs from biofluids, SEC struggles to resolve EVs from high concentrations of co‐isolates such as protein, nucleic acids, or lipoproteins present in these sources. Improved EV purity is important to allow exploration of the specific role of the EV‐linked mechanisms and the role of co‐isolates. To improve purity, repeated runs or multiple techniques need to be employed which are highly time consuming, laborious, and resource intensive and can ultimately result in lower EV recovery, defeating the purpose of upscaling.

To overcome these limitations of SEC, multimodal flowthrough chromatography (also known as bind‐elute chromatography or binding chromatography) was explored. Multimodal flowthrough chromatography (MFC) is an emerging method of EV purification that was first applied for purification of virus particles (James et al., 2016). The technique has risen to prominence in the EV field due to a lack of stringent purification techniques and numerous drawbacks associated with commonly used techniques, including operator dependent yields, EV deformities and aggregation, lack of purity and poor scalability (Nordin et al., 2019). Numerous studies for large biomolecule purification have reported MFC as a time‐efficient and scalable method that generates an intact product and consistently high EV yields (Corso et al., 2017; Lothert et al., 2020; Mertz et al., 2021).

Similar to SEC, MFC employs resin particles. However, unlike SEC resin, MFC resin includes an inert shell permeated with size‐selective pores that surround an absorptive core. Pores allow entry of free‐floating proteins and small molecules into the resin beads wherein they form hydrophobic and electrostatic interactions to the absorptive core. The pores are too small for large biomolecules and nanoparticles such as EVs, therefore allowing purified particles to be collected in the flow‐through.

Various groups have begun to explore the use of MFC for EV purification from 2D cell culture harvests (Corso et al., 2017; Nordin et al., 2019; Onódi et al., 2018; Zhou et al., 2023). One of the main benefits of MFC is its high scalability and high binding capacity. At larger scales, size separation effects between smaller and larger products may be seen during MFC, hence the technique is described as ‘multimodal’ despite its primary mode of purification being binding of impurities to the column. The high binding capacity and scalability of MFC potentially enables EV purification from harvests with high concentrations of impurities, including large‐scale EV harvests from 3D culture medium and biofluids. So far, the use of MFC for this purpose has not been explored. The primary aim of this study is to establish a comparison between the application of MFC and SEC to validate MFC as an alternative for the purification of EVs from large scale hollow fibre bioreactor harvests containing high concentrations of impurities. Moreover, this study encompasses an evaluation of MFC's performance against SEC across diverse sources for EV purification, including 2D cell cultures and human serum. The study includes comprehensive analyses of EV characteristics, particle and protein recovery rates, purity levels, and in vitro functionality to investigate the rationale behind utilising MFC for EV purification from a diverse range of sources.

## 2 MATERIALS AND METHODS

### 2.1 Cell culture

#### 2.1.1 3D bioreactor culture

HEK293T cells (ATCC) cultured in DMEM (Gibco) + 10% FBS (Gibco) + 1% 100x antibiotic/antimycotic solution (Sigma Aldrich) were expanded to 1 × 10^8^ cells in 15 cm dishes and seeded into the extra‐capillary space (ECS) of FiberCell Systems C2011 20 kDa molecular weight cut‐off (MWCO) hollow‐fibre bioreactor (FiberCell Systems). Once seeded, the glucose concentration of complete culture medium in the reservoir was checked daily, using a Bayer Contour XT Glucometer, to monitor the growth and metabolism of cells and to ensure an adequate supply of nutrients and glucose. When 50% of the starting glucose was depleted medium was renewed. For HEK293T cells 50% of the available glucose in 1 L of complete media was typically depleted in 48 h.

#### 2.1.2 2D culture

Cardiac progenitor cells (CPCs) were obtained as previously described (Smits et al., 2009). The human fetal heart tissue was obtained by individual written informed consent and after approval of the ethics committee of Leiden University Medical Center, The Netherlands according to the principles outlined in the Declaration of Helsinki for the use of human subjects or tissue. CPCs were cultured in MEM199 + Earle's Salts and L‐glutamine (Life Technologies) supplemented with 22% EGM‐2 medium (Lonza), 1% penicillin/streptomycin (Gibco), 10% fetal bovine serum (FBS) and 1% MEM NEAA Nucleic acids (Gibco). Human Microvascular Endothelial Cells‐1 (HMEC‐1) were cultured in MCDB‐131 medium (Life Technologies) supplemented with 10% FBS, 1% GlutaMAX (Gibco), 1% penicillin/streptomycin, 10 ng/mL rhEGF (Peprotech) and 50 nM Hydrocortisone (Sigma). All cells were cultured at 37°C and 5% CO_2_ in flasks or plates coated with 0.1% gelatin (Sigma).

For preparation of whole cell lysates (WCL), cells were collected in trypsin and centrifuged at 400 × *g* for 10 min. Cells were washed with PBS and collected in 1 mL complete lysis‐M Reagent (Roche) supplemented with protease inhibitor and phosphatase inhibitors. After incubation on ice for 30 min the solution was centrifuged for 10 min at 12,000 × *g* at 4°C. Supernatant was stored at −20°C.

### 2.2 EV purification

#### 2.2.1 3D bioreactor culture

To harvest conditioned medium from HEK293T cells cultured in the hollow‐fibre bioreactor and remove dead cells that had detached from the hollow fibres, DMEM (Gibco) supplemented with 1% antibiotic‐antimycotic solution (100×) (Sigma Aldrich) was flushed using a 50 mL syringe through the ECS from the left side‐port to a syringe on the right side‐port. A total of 22 mL of medium was then drawn into new syringes on each side port from the reservoir bottle, via the capillary pores. Withdrawn medium was flushed among the syringes four times to dislodge material within the ECS. This was repeated three times. Resulting harvests were pooled, centrifuged at 700 × *g* for 5 min, then 4000 × *g* for 20 min prior to further concentration. Centrifuged supernatant was concentrated to ∼5 mL by tangential flow filtration (TFF) with 100 kDa MWCO Sartorius Vivaflow 50R hydrosart filtration systems (Sartorius, Göttingen, Germany). Following TFF, concentrate destined for SEC was further concentrated to <2 mL with Amicon Ultra 100 kDa MWCO centrifugal filters (Merck Life Sciences) at 4000 × *g*. Subsequently, EV purification by size exclusion chromatography (SEC) was performed on an AKTA Pure (Cytiva, Marlborough, MA, USA) with a Tricorn 10/300 column packed with Sepharose Fast Flow 4 resin (Cytiva) to separate EVs from host cell protein. Samples destined for Capto Core multimodal flowthrough chromatography were not further concentrated following TFF. Concentrate was passed through either a 1 mL HiTrap 400, 1 mL HiTrap 700 or 5 mL HiScreen 700 Capto Core (CC700) column (Cytiva) and flow through was collected.

#### 2.2.2 2D culture

To purify CPC‐derived EVs, CPCs were washed with PBS when a confluency of 80%–90% was reached, and medium was replaced with supplement free MEM199 medium. Conditioned medium was removed after 24 h and centrifuged for 15 min at 2000 × *g*. Supernatant was filtered through a 0.45 mm PES bottle top filter (Nalgene). Filtrate was concentrated by TFF using a 100 kDa MWCO Minimate TFF capsule (Pall). During TFF, buffer exchange was performed with PBS. The concentrate was split equally and loaded onto a CC700 column or a Hiprep 16/60 Sephacryl S‐400 SEC column (Cytiva) connected to an ÄKTA start system (Cytiva). EV‐containing fractions were pooled and concentrated using a 100 kDa Amicon Ultra‐15 spinfilter (Merck).

#### 2.2.3 Serum

All blood samples were acquired from healthy consenting human donors by the Australian Red Cross Lifeblood. All procedures performed involving human participants were per the National Health and Medical Research Council guidelines and approved by the human ethics committee of La Trobe University. To reduce variability between runs, serum was pooled and centrifuged at 2000 × *g* for 10 min to remove larger particles and aggregates, and the resulting supernatant was aliquoted. The supernatant was removed, pooled together and mixed before being aliquoted into 500 µL aliquots and stored at −80°C. Samples were thawed prior to isolation and centrifuged at 10,000 × *g* for 10 min. The supernatant was immediately run through either a CC700 column or Tricorn 10/300 packed with Sepharose Fast Flow 4 resin connected to an AKTA Pure system (Cytiva). For each replicate, EV fractions from 3 runs were pooled and concentrated using a 10 kDa MWCO Amicon Ultra‐4 spinfilter (Merck).

### 2.3 Capto Q anion exchange

Samples destined for Capto Q anion exchange were subjected to multimodal flowthrough chromatography with prior incubation of 150 units/mL of benzonase (Merck Life Sciences) for 1 h at 37°C. The 1 mL Capto Q column (Cytiva) was primed with five column volumes of Tris HCl pH 8.0. Once purified by CC700 a 5 mL sample from the CC700 peak was taken and passed through a Capto Q column with Tris HCl pH 8.0 as the mobile phase. The elution step was conducted using Tris HCl pH 8.0 with a linear increase in NaCl concentration from 0 to 0.5 M over 15 column volumes. The NaCl concentration was finally increased to 1 M to strip any remaining material from the column. Samples from the flow through and elution phases were collected and pooled separately.

### 2.4 Western blot analysis and silver stain

For SDS PAGE (sodium dodecyl sulphate polyacrylamide gel electrophoresis), samples were diluted with lithium dodecyl sulphate sample buffer (ThermoFisher Scientific) and sample reducing agent (ThermoFisher Scientific), heated for 5 or 10 min at 95°C or 70°C, respectively, and separated on a 4%–12% Bis‐Tris polyacrylamide gel (Thermo Scientific) next to a PageRuler Plus Prestained Protein Ladder (ThermoFisher Scientific) or SeeBlue Plus2 Pre‐stained Protein Standard (Invitrogen) for serum samples. Proteins were blotted on a PVDF membrane and membranes were blocked for 1 h in 50% v/v Intercept Blocking Buffer (LI‐COR Biosciences) at room temperature (RT). Immune‐labelling was performed with 50% v/v Intercept Blocking Buffer overnight at 4°C or for 1 h at RT. Used antibodies are shown in Table 1. Imaging was performed on an Odyssey Infrared Imager (LI‐COR Biosciences) at 700 and 800 nm.

**TABLE 1:** Used antibodies during western blotting.

Antibodies	Brand	Dilution
Mouse anti‐alix	Thermo Scientific, MA1‐83977	1:1000
Rabbit anti‐alix	Abcam, ab186429	1:1000
Mouse anti‐alix	Abcam, ab117600	1:1000
Mouse anti‐syntenin	Origene, TA504796	1:1000
Rabbit anti‐syntenin	Abcam, ab133267	1:1000
Mouse anti‐flotillin	BD biosciences, 610820	1:1000
Mouse anti‐CD81	Santa Cruz, SC‐166029	1:1000
Rabbit anti‐annexin A1	Abcam, ab214486	1:1000
Rabbit anti‐calnexin	GeneTex, GTX 101676,	1:1000
Rabbit anti‐calnexin	Abcam, ab22595	1:1000
Mouse anti‐β‐actin	Sigma, A5441	1:1000
Rabbit anti‐β ‐actin	Sigma, A1978	1:1000
Rabbit anti‐TSG101	Abcam, ab30871	1:1000
Rabbit anti‐TSG101	Abcam, ab125011	1:500
Rabbit anti‐fibronectin FN1	Sigma, F3648	1:2000
Rabbit anti‐AKT	Cell Signaling Technology, 9272S	1:1000
Rabbit anti pAKT	Cell Signaling Technology, 4060S	1:1000
Rabbit anti‐MAPK (ERK1/2)	Cell Signaling Technology, 9102S	1:1000
Rabbit anti‐pMAPK (pERK1/2)	Cell Signaling Technology, 9101S	1:1000
Alexa Fluor 680 anti‐mouse	LI‐COR Biosciences, A‐21057	1:10.000
IRDye 800CW anti‐rabbit	LI‐COR Biosciences, 926–32211	1:7500
ECL Mouse IgG, HRP‐Linked	Cytiva, NA931‐1ML	1:10000
ECL Rabbit IgG, HRP‐Linked	Cytiva, NA934‐1ML	1:10000

Silver staining was performed to visualise total protein in differently purified samples. Gels were fixed for 1 h at RT in 40% ethanol (Fisher Scientific), 10% acetic acid (Fisher Scientific) in double distilled water followed by a 30‐min wash at RT in 50% ethanol. Subsequently, the gel was sensitised for 1 min in 0.02% sodium thiosulphate (Sigma Aldrich) solution followed by washing in two changes of double distilled water. The gel was stained for 30 min in 0.1% silver nitrate (Sigma Aldrich) solution then washed in two changes of double distilled water. Silver nitrate stain was developed in 250 mL of a 2% sodium carbonate (Sigma Aldrich), 0.04% paraformaldehyde (Sigma Aldrich) solution until desired staining was achieved. Development was stopped in 5% acetic acid solution for 15 min prior to rinsing in 2 changes of double distilled water for 5 min each.

### 2.5 Protein and nucleic acid quantification

Protein concentrations were determined using Micro BCA protein assay kits (Thermo Fisher Scientific) following the manufacturer's protocol. Nucleic acid concentrations were determined using the Quant‐iT RiboGreen RNA assay kit (Thermo Fisher Scientific) following the manufacturer's protocol.

### 2.6 Nanoparticle tracking analysis

Particle size and concentration was determined with a Nanosight NS500 (Malvern) using NanoSight NTA 3.3 software. Three 30‐s videos were recorded for each sample with a delay of 5 s between each video. For all the recordings, the camera level was set at 13–16 with a well‐adjusted camera focus for maximum sharpness. Detection threshold was set at 5, screen gain at 1.0 while other functions were set to automatic. Samples were diluted in PBS.

For serum derived EVs, size distribution and concentration were determined using a ZetaView PMX‐120 nanoparticle analyser (Particle Metrix) equipped with ZetaView Analyse Software version 8.05.12. Prior to measurement the system was calibrated as per manufactures instruction with 100 nm Nanospheres 3100A (ThermoFisher Scientific). Measurements were performed in scatter mode and for all measurements the cell temperature was maintained at 25°C. All samples were diluted in PBS to a final volume of 1 mL. Capture settings were: sensitivity 80, shutter 100 and frame rate 30. Post‐acquisition settings were: minimum trace length 10, brightness 30, area 5 and max area 1000.

### 2.7 Transmission Electron Microscopy (TEM)

EV solutions from HEK cells and CPCs were diluted with Milli‐Q water to a protein concentration of 100–200 µg/mL. Carbon coated 300 mesh copper grids were glow discharged and then inverted carbon side face down onto a 10 µL droplet of an EV suspension for 2 min. Subsequently, the grid was blotted with filter paper and stained for 10 s with 2% uranyl acetate, followed by blotting and air drying. Grids were imaged in a FEI Tecnai 12 transmission electron microscope (FEI Company, Hillsboro, OR, USA) at 120 kV using a Gatan OneView CMOS camera (Gatan, Pleasanton, CA, USA).

Isolated EVs from serum were imaged using a JEM‐2100 transmission electron microscope (JEOL Tokyo) equipped with a LaB6 filament operating at 200 kV. Images were recorded using a Gatan Orius SC200 2 k × 2 k charge‐coupled device camera at a range of magnifications. 400‐mesh carbon‐coated copper grids (ProSciTech) which had been glow‐discharged in the air to render them hydrophilic using an Emitech 950X equipped with a K350 glow discharge unit (Quorum Technologies) were used. Ten microlitres of the EV samples derived from serum following CC700 and SEC purification were dropped onto the prepared grids and left for at least 30 s. Excess fluid was drawn off with filter paper, and two drops of 2% Uranyl Acetate were added for approximately 10 s each before being drawn off with filter paper. The grids were then dried and transferred into transmission electron microscopy for viewing.

### 2.8 Endothelial cell wound healing assay

HMEC‐1 were seeded in a 48‐well plate at a density of 90,000 cells/well 48 h prior to the assay. A scratch wound was made using a pipet tip and detached cells were washed away with plain MCDB‐131 medium without any supplementation. Subsequently, cells were incubated in basal MCDB‐131 medium plus indicated treatments in triplicate for 6 h. Three micrograms of EVs per well was added as treatment, PBS was used as a negative control and MCDB‐131 containing 20% FBS as a positive control (Mol et al., 2017; Roefs et al., 2023). At *t* = 0 h and *t* = 6 h, two pictures per well were taken using an EVOS microscope (Life Technologies). Closing of the scratch was measured by image analysis using Image J software. The mean width of each scratch at *t* = 0 h was subtracted by the mean width at *t* = 6 h to determine the migrated distance. Relative wound closure was calculated relative to the negative control.

### 2.9 Endothelial signalling activation assay

For the endothelial signalling activation assay, HMEC‐1 cells were incubated with EVs and their lysates were used to measure the phosphorylation of AKT and ERK. HMEC‐1 cells were seeded in a 48 well plate at a concentration of 90,000 cells/well and incubated for 48 h. Then, the medium was washed and replaced with plain medium without any supplementation, and the cells were serum‐starved for 3 h in the empty medium. After 3 h, 3 µg of the EV treatments were added to the wells and PBS was used as negative control (Mol et al., 2017; Roefs et al., 2023). After 30 min, the medium was aspirated and the wells were washed with PBS. To lyse the cells, 100 µL complete lysis‐M buffer (Roche, Basel, Switzerland) including protease inhibitors (Roche) and phosphatase inhibitors (Roche) was added and incubated for 5 min on ice. Every well was scraped and the lysate was transferred to an Eppendorf tube. Samples were vortexed and centrifuged for 15 min at 12,000 × *g* at 4°C. Expression of AKT, ERK and their phosphorylated forms pAKT and pERK, were analysed by western blotting, normalized based on protein concentration. Used antibodies are shown in Table 1.

### 2.10 Apolipoprotein B ELISA

ApoB concentration of samples was quantified using an Apolipoprotein B quantikine ELISA (DAPB00, R&D Systems) following the manufacturer's protocol. The EV input was normalised based on volume.

### 2.11 Statistics

Statistical analyses were performed using GraphPad Prism 8.0. Differences between two groups were tested with a paired or unpaired *T*‐test. Comparisons of more than two groups were tested with one‐way ANOVA with Tukey's HSD multiple comparison post‐hoc test. In all cases values are reported as mean ± standard deviation, and *p*‐values <0.05 indicate statistical significance.

## 3 RESULTS

### 3.1 Size exclusion chromatography inefficiently separates EVs from co‐isolates in 3D culture conditioned harvests

**FIGURE 1:**
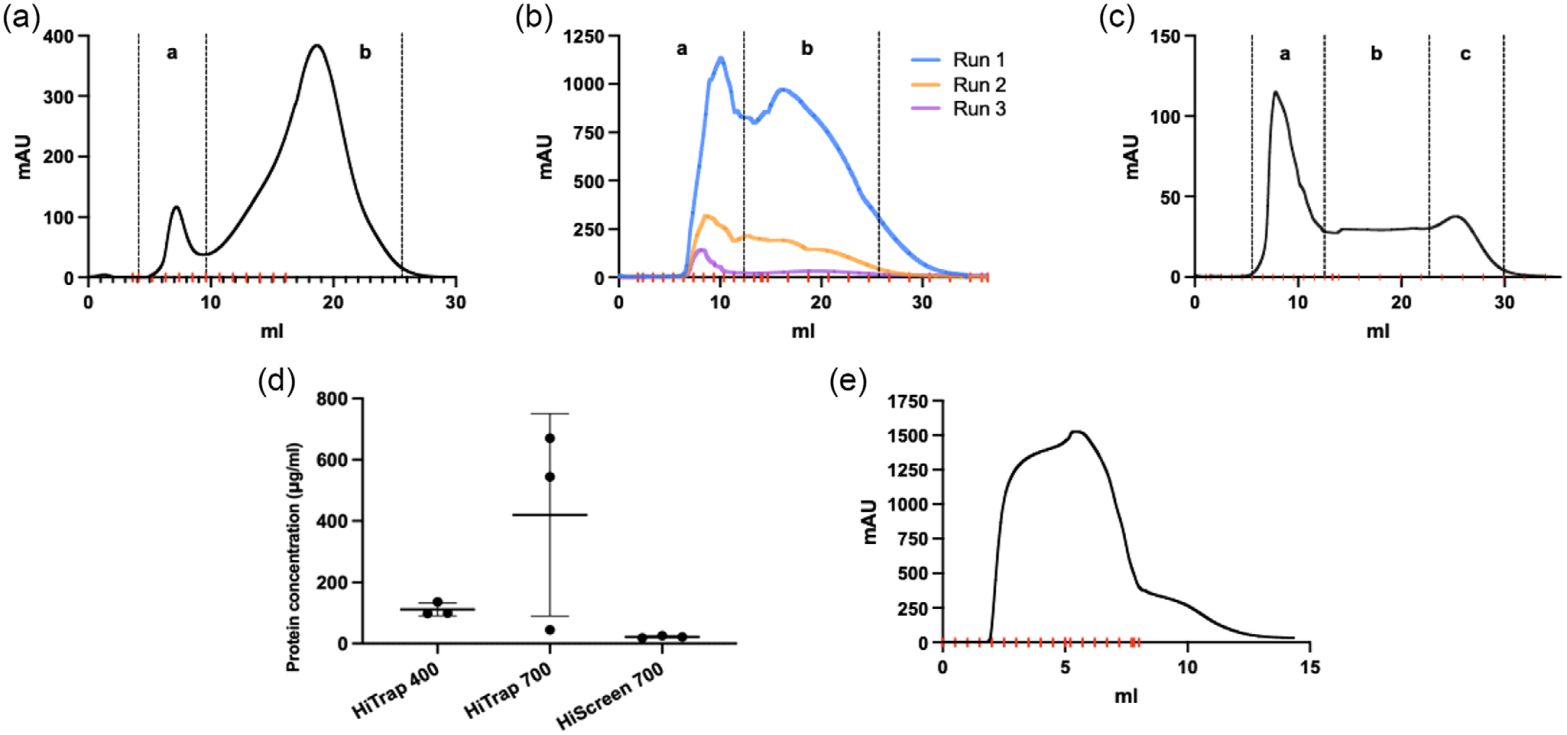
SEC cannot separate EVs from contaminants in bioreactor harvests, while MFC removes large quantities of free protein. (a) UV chromatogram of EVs purified with SEC from low scale 2D culture, where ‘a’ represents the EV/particle peak, and ‘b’ represents smaller contaminants such as free protein. (b) UV chromatogram of three consecutive passes of the same large scale bioreactor culture through SEC. (c) UV chromatogram of purified EV samples from the Capto Core purified by SEC to separate any remaining free protein or contaminants from the EV peak, where ‘a’ represents the EV peak, ‘b’ the contaminant peak, and ‘c’ represents the right shoulder attributed to DMEM constituents (Figure S1A). (d) Protein concentration in EV samples isolated using different Capto Core columns as determined by a microBCA protein assay. (e) UV chromatogram of a representative EV purification through CC700 MFC from large scale bioreactor culture.

As researchers encounter requirements to upscale EV production, concerns regarding whether SEC remains a capable method of EV purification have arisen due to the higher quantity of product and impurities that accompany the upscaling of EV production. Firstly, to establish the standard appearance of the chromatogram and the separation achieved when applying a low‐scale EV preparation to a SEC column, EVs from a low scale 2D culture harvest of HEK293T cells were purified by SEC using a Tricorn 10/300 column packed with Sepharose 4 Fast Flow resin. As shown in Figure 1a the resulting chromatogram from the SEC purification presents clear separation between EVs/particles (peak a) and contaminating soluble proteins and other impurities (peak b). Subsequently, to investigate whether SEC allowed for efficient separation of EVs harvested from large scale 3D culture, supernatant obtained from the HEK293T bioreactor was applied to the same SEC column. Figure 1b presents the resulting chromatogram where ‘Run 1’ represents the result of passing the concentrated bioreactor sample through the SEC column once (SECx1). Extensive crossover in both peaks was seen compared to 2D culture purification in Figure 1a. This indicated unsuccessful separation between EVs and co‐isolates. Purification of EVs could only be achieved after performing consecutive SEC runs on the peak‐a fractions. Three consecutive SEC runs were necessary to achieve complete peak separation (SECx3). However, there was a marked reduction in mAU in peak a; an early sign of potential reductions in EV yield. Taken together these results indicate the inefficiency of SEC to purify EVs from large scale 3D cultures.

Given the poor separation achieved by SECx1 and the added time needed to achieve complete separation between EVs and contaminants in SECx3, as well as indications of EV losses, an alternative method of EV purification from 3D culture was sought. Thusly, MFC using Capto Core columns was explored to determine whether MFC could yield EV preparations derived from bioreactor cultures that contain less co‐isolates than EVs purified by SEC. Prior to making comparisons to SEC, the most efficient MFC column was first identified. Three Cytiva Capto Core columns were compared; HiScreen 700, HiTrap 400 and HiTrap 700. Isolated fractions from the Capto Core columns were pooled and loaded on to a Tricorn 10/300 column to separate any remaining contaminants from EVs. Figure 1c presents a representative chromatogram from Tricorn 10/300 SEC following HiScreen 700 MFC. The chromatogram presented a characteristic EV peak (Figure 1c **‐a**), free protein/contaminant region (**b**) and a right shoulder (**c**). The right shoulder observed in the chromatogram was later attributed to components of DMEM and the used antibiotics. When DMEM + penicillin/streptomycin was injected into the SEC column, a peak was eluted in the same stage (Figure S1A). When medium or a high concentration of antibiotics were injected into a MFC column, it was also shown that these columns could not capture all the phenol red or antibiotics from the medium (Figure S1B–S1D). This did not cause any additional problems because TFF was subsequently able to efficiently remove phenol red and antibiotics from the media. The fractions from peak‐b were quantified by BCA assay to determine the free protein concentration remaining in Capto Core purified samples. Figure 1d shows that the HiScreen Capto Core 700 column was superior to the HiTrap 400 and HiTrap 700 columns, leaving just 21.74 µg/mL of free protein remaining in solution, ∼5x lower than the next best alternative the HiTrap 400 with 111.4 µg/mL remaining. As the best commercially available Capto Core column, the 5 mL HiScreen Capto Core 700 column (CC700) was selected for further comparison to SEC. The chromatogram presented in Figure 1e was acquired using a CC700 column for EV purification of concentrated bioreactor conditioned medium sample. The broad peak represents the entirety of the purified EV product, leaving protein impurities bound to the column. A shoulder on right side of the chromatogram demonstrated minor size separation effects.

**FIGURE 2:**
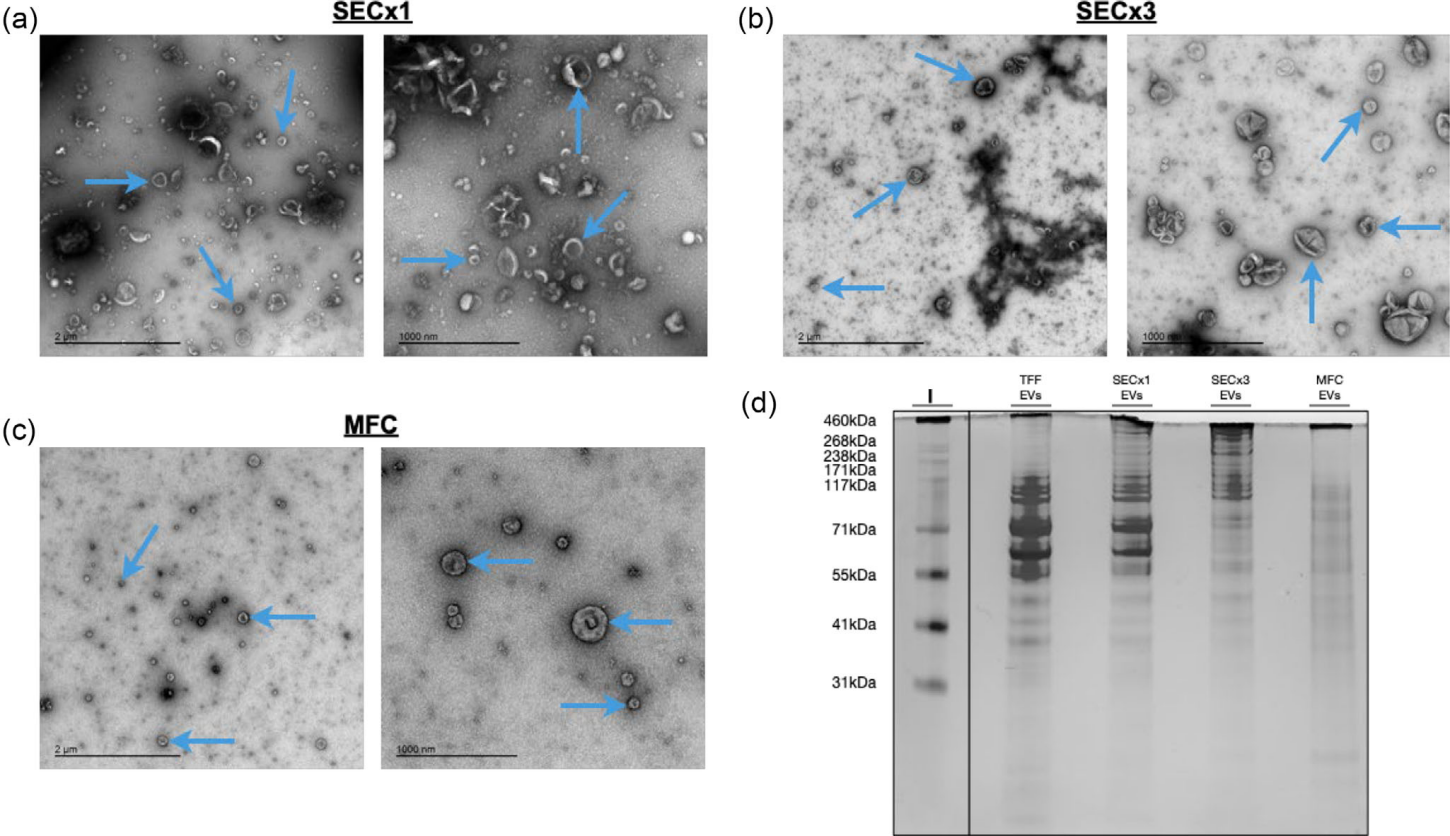
TEM images and silver staining present differences in constituents of MFC and SEC EV preparations. (a) TEM images from SECx1 and B SECx3 purifications. (c) TEM images from MFC purifications. The blue arrows indicate typical EV shaped particles. (d) Silver stain of TFF concentrated conditioned medium, SECx1, SECx3 and MFC samples. Equal total protein amounts (10 µg) were loaded.

Following identification of the HiScreen CC700 column as the most efficient MFC column for the removal of free proteins from bioreactor EV preparations, initial comparisons of EV purity between SEC and MFC were made. TEM analysis and silver staining were performed to investigate the EV purification efficiency of SECx1, SECx3 and MFC, and to determine the mass of proteins remaining within EV samples after SEC and MFC. TEM analysis of SECx1 and SECx3 samples (Figure 2a,b) revealed spherical, membrane‐encapsulated particles, characteristic of EVs. However, many dark stained co‐isolates were also clearly seen in both samples, but with arguably more in SECx1. While somewhat cleaner, extensive EV aggregation was seen in SECx3 samples though less present in SECx1. In contrast, dark staining co‐isolates were markedly less apparent in MFC TEM images (Figure 2c) allowing EVs to be observed more clearly. Furthermore, EVs appeared notably less aggregated in MFC samples and EVs appeared more spherical. Silver staining (Figure 2d) was performed to provide a visual representation of the remaining protein in differently purified samples. TFF concentrated conditioned medium and SECx1 samples were highly saturated in proteins greater than 55 kDa. Compared to SECx1, SECx3 removed most of the contaminant proteins between 55 and 171 kDa, however a high concentration of proteins greater than 171 kDa were also copurified with EVs. Comparatively, MFC removed all traces of high molecular weight proteins leaving a uniform stain.

### 3.2 Multimodal flowthrough chromatography yields EV preparations with lower protein co‐isolates and higher recovery rates than size exclusion chromatography when purifying EVs from large scale cultures

**FIGURE 3:**
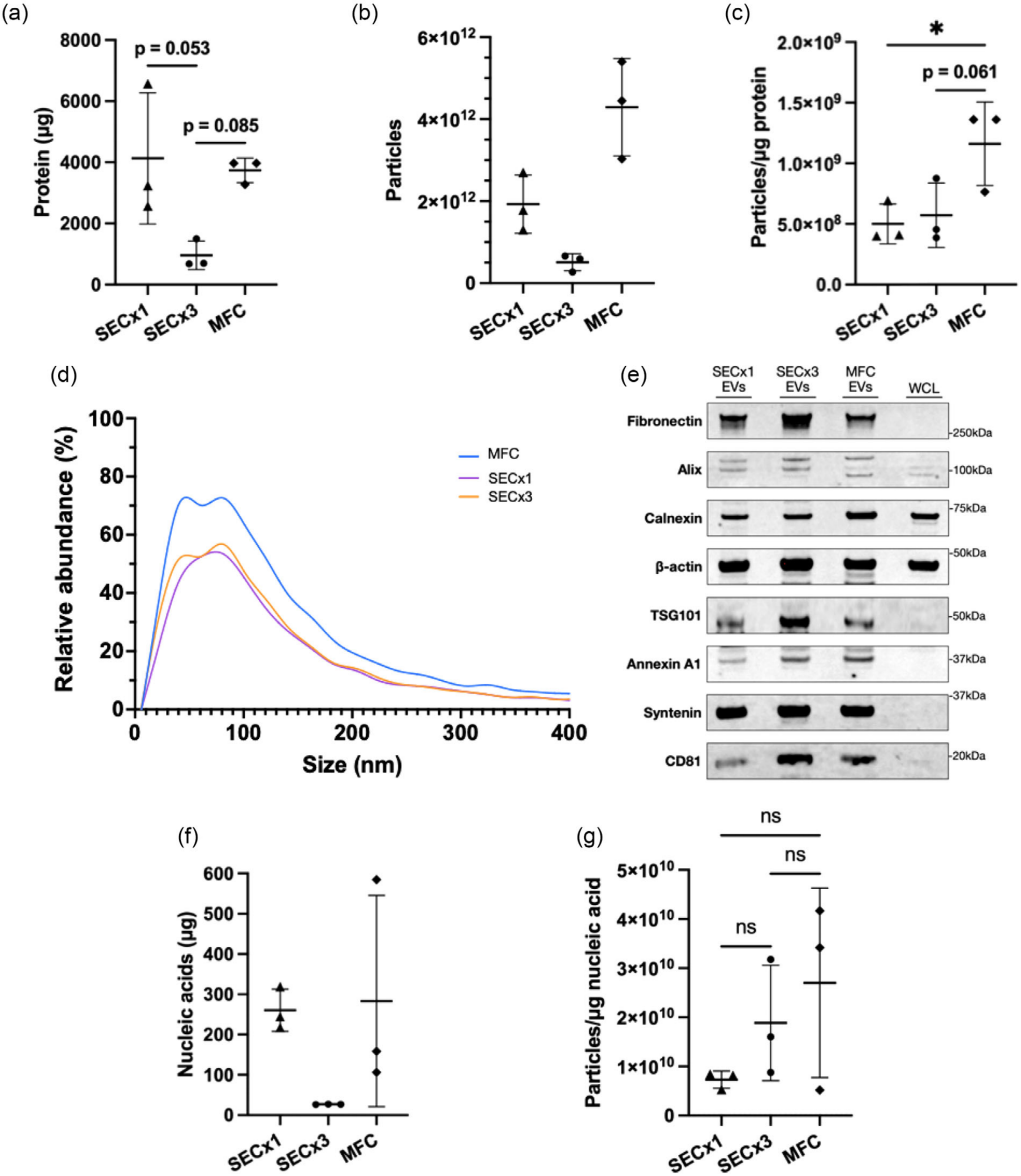
Multimodal flowthrough chromatography presents a significant improvement in EV purity and yield versus SEC. (a) Protein yields as determined by a microBCA protein assay. (b) Particle yields determined by NTA. (c) Particle/protein ratios of isolates. (d) Particle size distributions determined with NTA. (e) Western blot analysis of EV proteins alix, β‐actin, syntenin, CD81, annexin A1 and TSG101. Equal protein amounts (10 µg) were loaded. (f) Nucleic acid yields determined by Quant‐iT RiboGreen RNA Assay. (g) Particle/µg of nucleic acid ratios. Results in B, C, D, E, F and G represent biological replicates (*n* = 3). Significance levels are indicated with asterisks (*p < 0.05).

After preliminary comparisons of EV purity between bioreactor SEC‐ and MFC‐preparations, total protein, particle yield and particle‐protein ratios were determined and compared to gain further insights into relative purities of EV preparations following SEC and MFC. TFF concentrated 3D preparations were purified by MFC or SEC and analysed by BCA assay and NTA. As shown in Figure 3a, total protein in MFC purified samples remained almost as high as in SECx1 samples. On the other hand, SECx3 total protein was over four times lower. Total particles yielded after MFC purification was approximately eight times higher following MFC purification than SECx3 and 2x higher than SECx1, respectively (Figure 3b). Therefore, particle/protein data demonstrated a trend in the data towards a higher purity of MFC purified EVs than SECx3, and a significantly higher purity of MFC purified EVs than SECx1 (Figure 3c). Figure 3d displays the size distributions of differentially purified particles. The majority of particles obtained from SECx1, SECx3 and MFC were smaller than 200 nm, which is typical of small EVs (Théry et al., 2018), with a large overlap in their distribution. To further characterise differentially purified particles, various proteins were analysed by western blot (Figure 3e). High expression of CD81, alix, TSG101, syntenin and annexin A1 in SEC and MFC samples versus low to no detectable expression of these markers in HEK whole cell lysate was characteristic of EVs. A leap in EV purification between SECx1 and SECx3 was visualised as unanimous increases in EV marker expression in SECx3. SECx3 and MFC protein expression was very similar, suggesting that MFC was able to purify EVs at least to the same extent as SECx3, however a higher expression of the extracellular matrix protein, fibronectin, was seen in SECx3 samples compared to MFC, suggesting that SEC remained unable to fully purify EVs from co‐isolated proteins. Moreover, calnexin remained more present in MFC samples than in SECx3 samples. Altogether, MFC yielded purer bioreactor culture‐derived EVs than SEC, moreover, by MFC higher particle yields were obtained that retained typical EV characteristics.

After establishing the ability of the MFC columns to deplete free protein, whether MFC could deplete free nucleic acids from bioreactor harvests was assessed using Quant‐iT RiboGreen assays on purified EV samples. Figure 3f presents SECx3 as the method that contained the least nucleic acid co‐isolates from bioreactor harvests. Despite large standard deviation, MFC yielded the most remaining nucleic acids, closely followed by SECx1. Both methods presented total nucleic acids levels of over 250 µg. However, pertinently, MFC yielded higher total particles than SEC, therefore the quantity of remaining nucleic acids per purified particle was lower compared to SEC (Figure 3g). In order to reduce free nucleic acids co‐isolates, we evaluated the use of benzonase, a nuclease which breaks down nucleic acids. After incubating TFF concentrated harvests with 150 units/mL benzonase prior to MFC purification, nucleic acids concentrations were reduced to 165.9 µg (Figure S2A). In addition to benzonase, anion exchange (AIEX) using a Cytiva Capto Q column was tested for further removal of nucleic acids. Purified EVs from MFC were applied to the AIEX column and flowthrough fractions were tested for the nucleic acid and particle concentrations. The flowthrough of the AIEX column showed reduced levels of nucleic acids but resulted also in a reduced number of particles (Figure S2A and S2B). The reduction in particle yield was however relatively smaller, thereby resulting in a greater number of particles per microgram of nucleic acids (Figure S2C). NTA size distribution of particles from AIEX purification showed a typical EV size range, however, AIEX appeared to remove part of a population of sub‐60 nm particles seen in other runs, demonstrated by the lack of a bimodal peak seen in other distributions (Figure S2D). TEM images presented highly pure ‘cup‐shaped’ spherical EV particles derived from AIEX flowthrough, demonstrated by a distinct lack of other structures (Figure S2E). Far fewer EVs were seen from the elution phase of AIEX purification demonstrating that the majority of EVs were eluted, as expected, in the flow through steps. A feint expression of most EV proteins (alix, β‐actin, syntenin, CD81, annexin A1 and TSG101) was visible in AIEX western blot samples likely resulting from a marked loss of EVs compared to MFC purification (Figure S2F). Calnexin and fibronectin appeared to be further reduced in AIEX samples.

In conclusion, both benzonase and AIEX treatments reduced nucleic acid levels, but reductions in EV concentration presented a notable trade‐off to achieve this outcome. Substantial reductions in total particle counts and EV marker expression highlighted the need for optimisation of anion exchange following MFC to retain high EV concentrations.

### 3.3 Multimodal flowthrough chromatography purifies EVs from 2D cultures with higher recovery rates and preserved function compared to size exclusion chromatography

In addition to large scale bioreactor culture, MFC was evaluated for EV purification from 2D cultures and compared to SEC to establish whether MFC provided any improvement to EV purity, yield or functionality when using smaller scale cultures. CPCs were used as a cell source to generate EVs to compare EV functionality following MFC and SEC. Conditioned medium from 2D culture of CPCs was consecutively centrifuged, filtered and concentrated by TFF, then equally divided and loaded onto either a CC700 MFC column, or Hiprep S‐400 SEC column to purify EVs. Following elution, EVs were concentrated, and EV purity, concentration, yield and in vitro functionality were determined.

**FIGURE 4:**
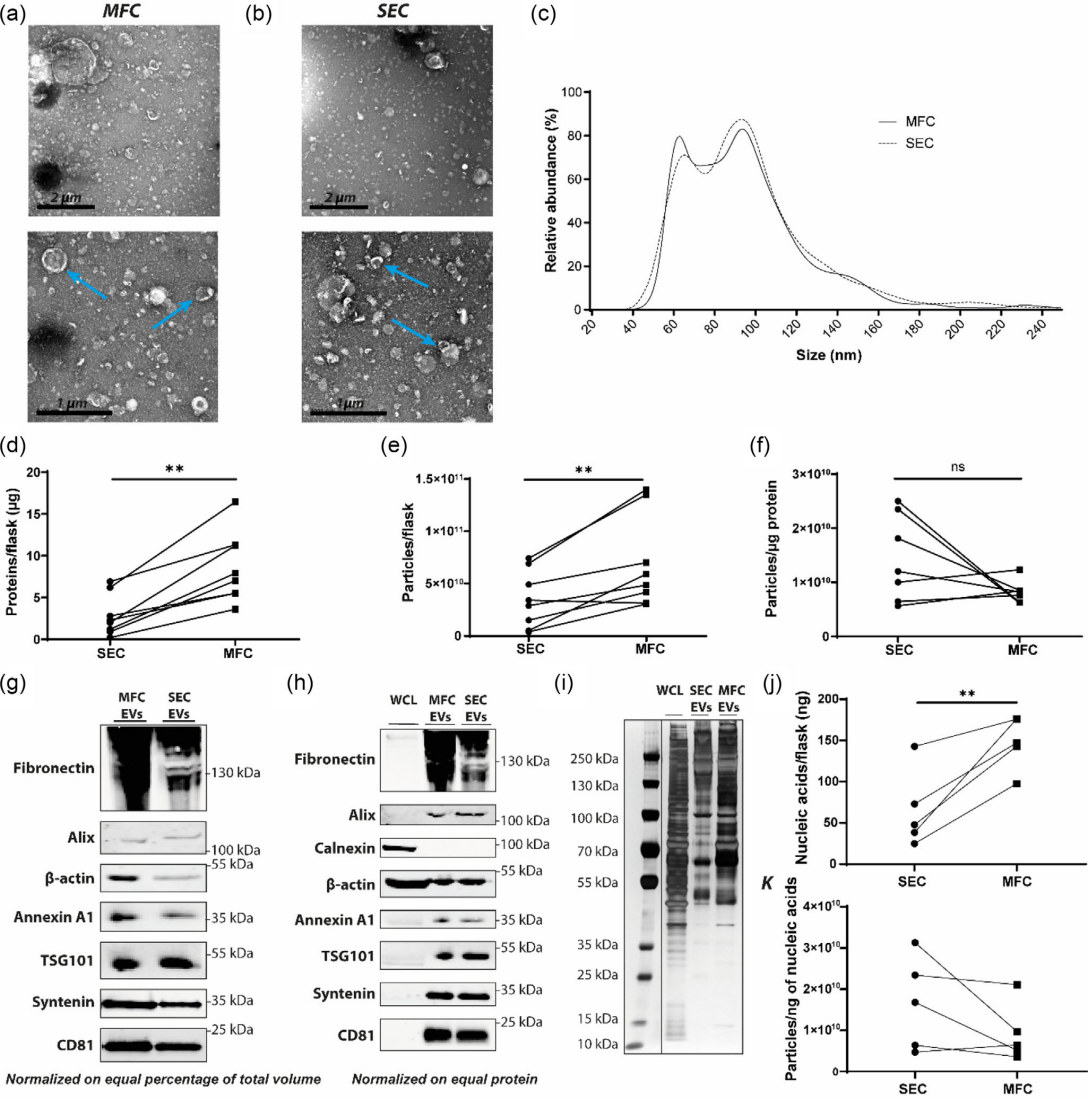
MFC allows EV purification from 2D culture with higher recovery rates and similar purity compared to SEC. TEM images of EVs purified with MFC (a) and SEC (b). The blue arrows indicate typical EV shaped particles. (c) Particle size distributions determined with NTA. (d) Protein yield as determined by a microBCA protein assay normalised per T175 flask. (e) Particle yield determined by NTA normalised per T175 flask. (f) Particle/protein ratios. (g) Western blot analysis of EV markers fibronectin, alix, β‐actin, annexin A1, TSG101, syntenin and CD81. Equal percentages of the total volume of EV isolates were loaded. (h) Western blot analysis of EV markers fibronectin, alix, calnexin, β‐actin, annexin A1, TSG101, syntenin and CD81. Equal protein amounts (2 µg) were loaded. (i) Silver stain analysis of EVs and a WCL. Equal protein amounts (2 µg) were loaded. (j) Nucleic acid yields per T175 flask as determined by a Quant‐iT Ribogreen assay. (k) Particle/ng of nucleic acid ratios. Results in C, D, E, F, G and K represent biological replicates (*n* = 4–8). Significance levels are indicated with asterisks (**p < 0.01).

The purity and integrity of EVs purified by SEC and MFC from 2D cultures was first assessed by TEM. Figure 4a,b present TEM images of bilayer‐enclosed particles with spherical morphology from both SEC and MFC, with no notable differences observed between particles purified by either method. Furthermore, analysis of EV size distribution by NTA revealed no apparent differences between particles purified by either SEC or MFC (Figure 4c). In contrast to the similarities seen in TEM and NTA, 2D culture preparations purified by MFC presented a significantly higher protein and particle recovery than preparations purified by SEC (Figure 4d,e), however no significant difference in particle/protein ratio was observed (Figure 4f). While some SEC purifications presented higher particle/protein ratios than MFC, overall SEC particle/protein ratios were far more variable than MFC which seemed more reproducible in terms of purity. Together these data suggested that MFC consistently produced at least equivalently pure EVs to SEC from 2D culture, but with significantly increased particle numbers.

Next, differences in the protein contents of preparations purified from 2D cultures by SEC and MFC were characterised by western blotting and silver staining. Following blotting of equal percentages of the total volume of EV isolates, analysis of the expression of EV marker protein revealed higher recovery rates of these markers in MFC‐ than SEC‐purified samples (Figure 4g). Validation of EV purity was performed via western blot analysis, normalized by equal amounts of protein (Figure 4h). Both MFC and SEC purified EVs presented a higher expression of EV marker proteins compared to total cell lysate. Only calnexin, which is a negative marker of EV purity, and β‐actin, which is highly abundant in cells, were more highly expressed in the cell lysates. Expression levels of EV markers were comparable for both MFC and SEC, indicating that EVs isolated by both methods were of similar in purity. Fibronectin was the only marker to be present in higher levels in MFC purified EVs compared to SEC. Upon comparing total protein profiles by silver staining, small differences in band intensities were observed between MFC and SEC samples (Figure 4i). Overall, few differences in EV marker expression and total protein profile were seen, presenting MFC as comparable to SEC for EV purification from 2D culture in terms of purity, however, producing significantly higher particle yields.

Finally, nucleic acid levels in EV samples purified from 2D cultures by SEC and MFC were compared to assess the capability of SEC and MFC to deplete the nucleic acid content of 2D culture preparations. The nucleic acid co‐isolated in EV samples from 2D culture was far lower compared to bioreactor isolates. This suggested that nucleic acid contamination is less prominent in 2D culture compared to 3D culture. Similar to the bioreactor purifications, higher nucleic acid levels were observed in the MFC purified EVs (Figure 4j). Nevertheless, nucleic acid levels presented a trend similar to the particle and protein counts of EVs, thus did not result in a difference in the total particle/nucleic acid ratios (Figure 4k).

**FIGURE 5:**
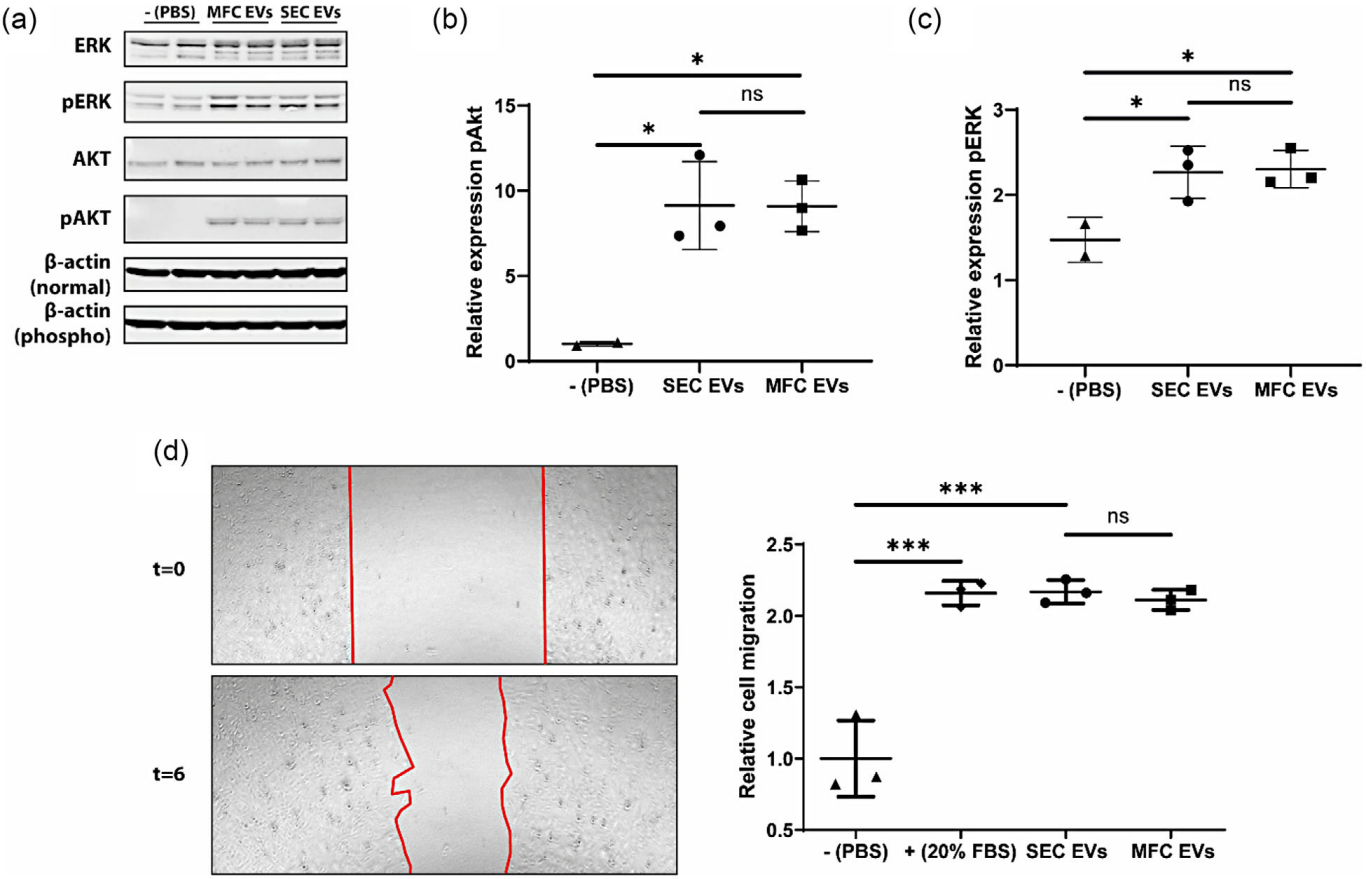
MFC and SEC isolated EVs have similar biological activity. (a) Results of an endothelial ERK and AKT phosphorylation assay performed with EVs purified by SEC and MFC. (b) Quantification of the relative pAKT expression to AKT. (c) Quantification of the relative pERK expression to ERK. (d) (Left) Example images showing the wound healing quantification and (right) the quantified results of an endothelial cell wound healing assay performed with EVs purified by SEC and MFC. Different biological replicates were used for the scratch assay (*n* = 3). Significance levels are indicated with asterisks (*p < 0.05, ***p < 0.001).

The most important remaining parameter to be tested is EV functionality, which was previously reported to be variable between different EV separation methods (Mol et al., 2017; Whittaker et al., 2020). To evaluate whether MFC and SEC isolated CPC‐derived EVs differed in their biological activity, AKT and ERK phosphorylation, and a wound healing migration assay were performed. AKT and ERK are intracellular signalling proteins involved in cell proliferation, angiogenesis, differentiation, adhesion, migration and survival (Ballif & Blenis, 2001; Karar & Maity, 2011). Confluent cultures of endothelial cells were treated with EVs purified by MFC or SEC, and the phosphorylation ratio of ERK and AKT was analysed by western blotting (Figure 5a). Treatment with EVs purified by either method led to significantly higher phosphorylation of AKT and ERK compared to the negative control (Figure 5b,c). No difference in phosphorylation was seen between treatments with EVs purified by SEC or MFC. For the wound healing assays, a scratch was made in a confluent endothelial cell monolayer, and the wounded monolayers were treated with EVs purified by SEC or MFC. Migration properties were determined after 6 h of treatment (Figure 5d). Both EVs purified with MFC or SEC significantly increase endothelial cell migration compared to the PBS negative control.

Altogether, isolation of EVs from 2D culture by MFC yielded EVs of a similar purity to SEC but with significantly higher particle yields. Importantly, MFC and SEC isolated EVs did not differ in their biological activity.

### 3.4 Multimodal flowthrough chromatography isolates EVs from human serum with similar purity and higher recovery rates compared to size exclusion chromatography

**FIGURE 6:**
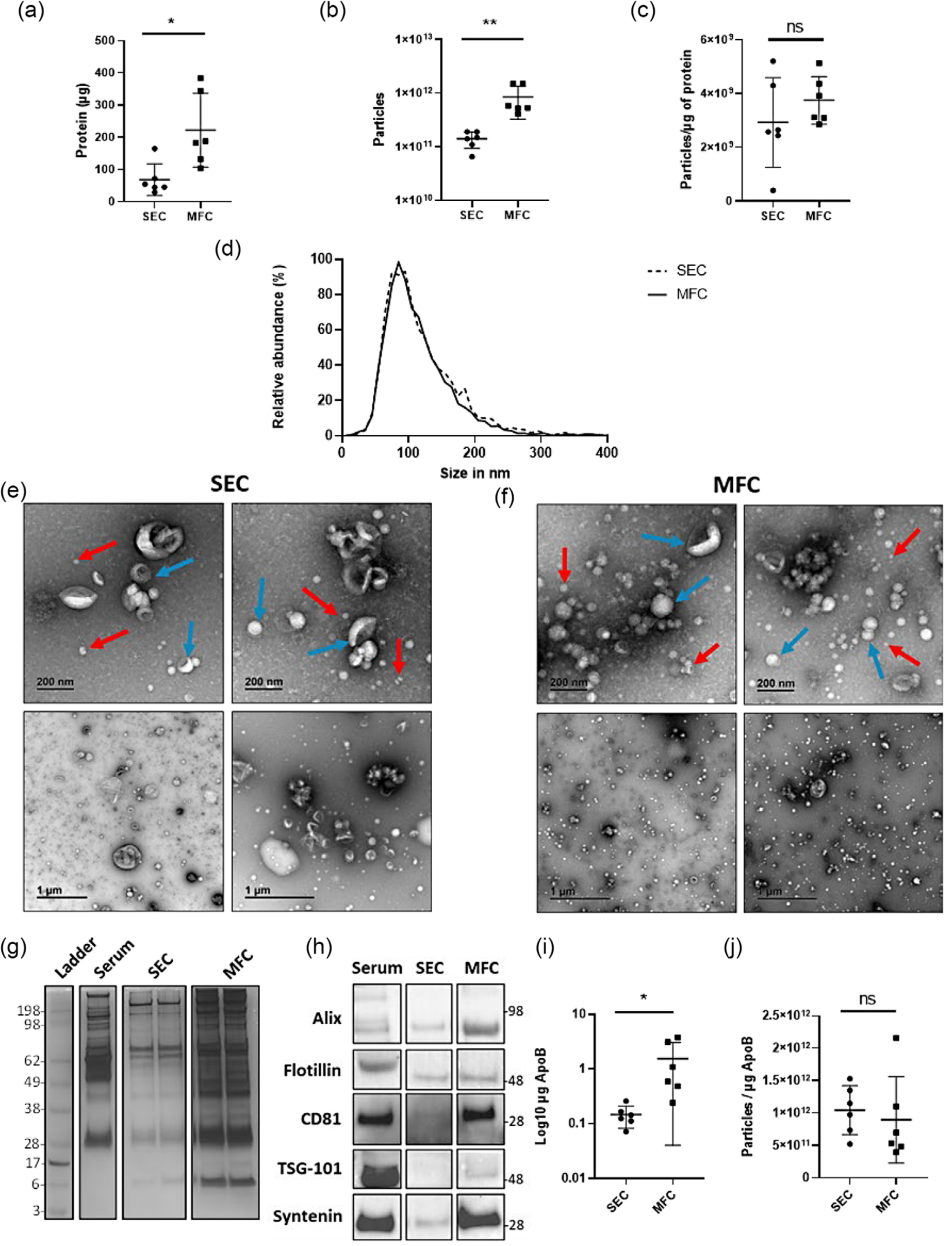
MFC exhibits a higher recovery rate of EVs from human serum than SEC while maintaining a similar level of purity to SEC. (a) Protein yields as determined by a microBCA protein assay. (b) Particle yields determined by NTA. (c) Particle/protein ratios of EVs. (d) Normalised mean particle size distributions determined with NTA. (e) TEM images of serum isolates from SEC. (f) TEM images of serum isolates from MFC. Red arrows indicate small particles of 60 nm or less. The larger blue arrows indicate typical EV shaped particles. (g) Silver stain analysis of crude serum, serum EVs isolated by SEC, and serum EVs isolated by MFC. Equal protein amounts (2 µg) were loaded. (h) Western blotting of EV‐associated proteins alix, TSG101, CD81, flotillin and syntenin in both SEC and MFC isolated samples loaded to equal volume. (i) Apolipoprotein B detected with ELISA. (j) ApoB/particle ratio. Results in A, B, C, D, I and J represent biological replicates (*n* = 6). Significance levels are indicated with asterisks (*p < 0.05, ***p < 0.001).

MFC was further evaluated to assess its capability to purify EVs from lipoprotein rich biological samples compared to SEC. 500 µL of 2000 × *g* centrifuged serum was used for EV purification by either SEC or MFC. Following BCA analysis of each EV preparation, more total protein was detected in MFC samples compared to SEC (Figure 6a). Next, particle concentration and size were determined using Zetaview NTA. Significantly more particles were detected from MFC purified samples compared to SEC (Figure 6b), although particle/protein ratio presented no significant difference in purity of EVs (Figure 6c). This suggested that the relative purity of both methods is comparable, but MFC yielded a greater number of particles. Additionally, NTA analysis revealed that EVs isolated from serum by either SEC or MFC demonstrated similar size distributions (Figure 6d). TEM analysis were conducted to confirm the size and morphology of serum‐derived EVs derived and presented approximately 100 nm particles with spherical morphology in both samples, with more particles present in MFC isolates than SEC, aligning with the NTA observations. Additionally, several particles smaller than 60 nm that are below the limit of detection of NTA were visible in both SEC and MFC isolates (Figure 6e,f).

Finally, the result protein content of serum‐derived EVs purified by SEC or MFC was characterised by silver staining and western blotting, and lipoprotein content was assessed using an apoB ELISA. As shown in Figure 6g, the presence and absence of different bands in the 98 and 49 kDa region following silver staining presented differences between EVs purified by SEC and MFC (Figure 6g). Additionally, western blot analyses revealed that samples purified by both SEC and MFC presented differences in expression of EV‐associated proteins. CD81 was enriched in MFC samples, while syntenin and flotillin was found in both SEC and MFC isolates. Furthermore, Alix was detected in all isolations but more strongly in MFC, while TSG‐101 was only detected in MFC and not in SEC. These results suggested that MFC elicited a greater EV yield than SEC due to the higher intensity of EV‐associated markers in MFC preparations. The lack of detectable markers in SEC samples may be due to low protein content in SEC isolates (Figure 6h, complete images are shown in Figure S3A–E). Finally, an apoB ELISA was conducted to investigate isolation purity further and evaluate the low‐density lipoprotein (LDL) co‐isolated by each method. Lower levels of apoB were detected in SEC purified samples compared to MFC (Figure 6i). However, when apoB content was normalised to particle count, no significant difference was observed (Figure 6j).

Together, this data demonstrates that MFC is a useful technique to isolate EVs from serum. Compared to SEC, MFC resulted in higher EV recovery and presented as more readily callable. However, both SEC and MFC suffer from co‐isolation of contaminating lipoprotein, and neither method was found to be better at depleting apoB.

## 4 DISCUSSION

Upscaling EV production to attain high EV concentrations is often a necessity when studying EVs, and will be paramount to the clinical development of EV therapeutics. Upscaling is often achieved using large scale cell culture techniques such as 3D bioreactors‐, suspension‐ or 2D cell cultures. However, with high EV yields come high concentrations of co‐isolates such as host cell proteins and nucleic acids. Consequently, conventional EV purification methods such as SEC are placed under high strain to produce pure EV preparations. Besides upscaling production, higher EV concentrations can also be reached with the improvement of the EV recovery rates during downstream purification methods. It has been shown that using SEC as a purification method could cause EV losses up to 80% (Zhang et al., 2020). MFC has emerged as potential alternative to SEC for EV purification (Corso et al., 2017) by binding small impurities such as proteins via electrostatic and hydrophobic interactions. Larger EVs cannot enter the inert size exclusive bead cores and will elute. Because EVs directly elute from the MFC columns, the EVs are less diluted compared to SEC. Furthermore, due to the high binding capacity and the flow through attribute, larger volumes of sample can be loaded on the column without hampering the resolution of the separation, which is the case when larger sample volumes are loaded on the SEC. This property makes MFC more suitable to scale up EV purifications. Here, we aimed to compare the use of SEC to novel Capto Core MFC, as an alternative approach to purify EVs derived from large scale bioreactor culture and standard scale 2D culture. Furthermore, MFC was also explored for the purification of EVs from serum biofluids. EV yields and characteristics were compared from both purification methods to study the suitability of MFC to purify EVs from these different sources.

We first assessed the ability of Capto Core MFC columns to purify EVs from bioreactor culture preparations and compared the purification efficiency to SEC. Whereas SEC produced a good separation of EVs from co‐isolates from 2D culture preparations, it was not suitable to obtain pure EVs from bioreactor cultures. High protein background was seen in SECx1 samples, and successive SEC runs of bioreactor preparations (SECx3) were required to attain complete resolution between EVs and co‐isolates. In contrast, a single MFC run was able to purify EVs from large scale bioreactor cultures. The HiScreen Capto Core 700 (CC700) column elicited the greatest reduction in total co‐isolated protein compared to other Capto Core columns and thus was selected for further comparison with SEC. Most other studies that tested Capto Core columns also chose the CC700 for EV purification (Corso et al., 2017; Nordin et al., 2019; Onódi et al., 2018; Reiter et al., 2019). McNamara et al. was the only group to use the smaller HiTrap Capto Core 700 column (McNamara et al., 2018). TEM images and particle/protein ratios presented EVs preparations yielded by MFC to contain less co‐isolates than SECx1 and SECx3. Furthermore, EVs in SEC images appeared to be more aggregated and warped. This was interesting as previous studies did not report EV aggregation or changes in morphology upon SEC isolation (Benedikter et al., 2017; Brennan et al., 2020; Nordin et al., 2015; Tzaridis et al., 2021). This phenomenon is more typical of differential ultracentrifugation where high shear forces cause aggregation and rupture of EVs (Linares et al., 2015; Vader et al., 2016). As opposed to a feature of SEC, it is possible that due to the high EV and co‐isolation concentrations within bioreactor culture preparations, high pressure may be exerted on EVs when attempted to be purified by SEC due to exceeding the maximum load of the column, therefore causing aggregation. The high, but similar expression levels of EV markers provided further evidence for the presence of highly pure EVs in both MFC and SECx3 (Théry et al., 2018). Interestingly, calnexin expression was also observed in all SEC and MFC samples. This was surprising as historically calnexin has been considered a non‐EV associated marker (Lötvall et al., 2014). However, more recent studies that have separated cell derived particle preparations into their respective constituents using density gradient ultracentrifugation have detected calnexin in EV associated fractions, including in particle preparations derived from HEK293T cells. These studies have shown that calnexin is not as underrepresented as other non‐EV proteins more usually associated with nuclear and mitochondrial processes and post‐translational modification (Choi et al., 2022; Kugeratski et al., 2021). Furthermore, calnexin has also been suggested as a marker of large EVs (>150 nm) (Théry et al., 2018). In contrast, calnexin expression observed here may also suggest the presence of non‐EV particles such as protein aggregates that are too large to be excluded by SEC or captured by MFC (Jeppesen et al., 2019). Other studies have reported a low expression of calnexin from SEC and MFC purifications by western blotting (Chen et al., 2020; Saludas et al., 2022), although expression is dependent on cell source and can be influenced by culture methodology as presented by Jakl et al. (2023) who showed differences in EV membrane protein expression between 2D and 3D culture in hollow fibre bioreactors (Jakl et al., 2023). Additionally, the greater fibronectin band intensity in the SECx3 sample may indicate a greater purity of MFC EVs than SECx3 EVs as fibronectin is a secretory protein not typically associated with EVs and only associates with EVs by binding to promiscuous receptors on the EV surface (Foers et al., 2018; Théry et al., 2018).

Although MFC had more EVs per µg of nucleic acids than SECx3, nucleic acids remained highly abundant in MFC purified bioreactor cultures. The source of these nucleic acids may be the higher quantity of dead cells produced in the bioreactor, as cells within hollow fibre bioreactors do not require splitting or passaging, and are grown in a post‐confluent manner with a continuous supply of fresh nutrient supplied through the hollow fibres. Alternatively, remaining nucleic acids might be directly associated with EVs, either luminally or associated with RNA binding proteins on EV surfaces (Di Liegro et al., 2017). Nonetheless, solutions to further reduce total nucleic acid levels and increase EV purity were explored. Interestingly, treatment with benzonase resulted in a reduction in nucleic acids but was accompanied by a reduction in total particles (Figure S2B). Total particle losses may have been due to damage to EVs following digestion of any EV associated nucleic acids, or due to degradation after the 1‐h incubation step at 37°C. Another possibility would be the presence of co‐isolated nucleosome particles containing nucleic acids, which are degraded by benzonase (Buzas, 2022; Thierry et al., 2016). However, other studies that have investigated this did not report a loss of EVs or change in EV integrity (Galbiati et al., 2021; Liu et al., 2022). AIEX presents a potential alternative to further reduce nucleic acid levels and yielded the highest particle per µg of nucleic acid purity but caused a marked reduction in EV yield (Figure S2C). Further exploration and optimisation of anion exchange will be necessary to reduce nucleic acid co‐isolation while retaining high EV concentrations.

Following observations of an improved EV purification from bioreactor cultures, MFC‐EV purification from 2D culture was compared to SEC to determine whether MFC could also yield higher EV yields or purer EV preparations from cultures with lower co‐isolate densities. As shown in TEM images, particle/protein ratios and western blotting, no major differences in purity were observed between MFC and SEC. Only fibronectin gave a higher expression in MFC purified EVs compared to SEC, which could indicate a greater purity of EVs isolated with SEC. In contract to the bioreactor culture, calnexin could not be detected in the EV isolates from 2D culture using either isolation method. While calnexin is often looked at as a negative ‘exosome’ marker, calnexin, as mentioned above, has been shown to be present in other EV subtypes (Crescitelli et al., 2020; Jakl et al., 2023; Lázaro‐Ibáñez et al., 2019; Marassi et al., 2021). Our data suggest that the culture platform influences relative abundance of the different EV subtypes released. EV preparations purified with both columns also had a similar particle/nucleic acid purity. Unlike EVs purified from bioreactor cultures, 2D cultures gave considerably lower nucleic acid contamination (around 1000‐fold). While it is shown here that SEC has problems purifying EVs from cultures with a higher concentration of impurities, SEC is able to isolate EVs with high purity from 2D cultures. Interestingly, significantly higher protein and particle recovery was seen using the MFC compared with SEC.

In the examination of EV isolation methods, it is crucial to validate the preservation of EV functionality (Paolini et al., 2022), as different EV purification methods have been shown to affect their functional attributes (Mol et al., 2017; Whittaker et al., 2020). An AKT and ERK activation assay and a wound healing migration assay were performed since EVs derived from CPCs have been shown to activate endothelial cell signalling pathways and migration (Mol et al., 2017; Vrijsen et al., 2016). For the performed functional assays, EVs purified from both columns were equally functional, indicating that MFC did not impair EV functionality compared to SEC.

Altogether, MFC presented as a viable alternative to SEC. In 2D cultures, MFC has no advantage over SEC related to EV purity, but it gave higher EV recovery rates with preservation of EV functionality.

In addition to bioreactor and 2D cell culture, MFC was also compared to SEC for EV isolation from serum due to the difference in composition of biofluids to cell culture medium. MFC yielded higher particle and protein yields than SEC with no significant change in the particle/protein ratio. Therefore, MFC produced higher particle yields without a significant loss in purity. However, the size and presentation of some particles recovered by SEC and MFC from serum samples were synonymous with reports of lipoprotein particles in EV preparations (Huang et al., 2021). These findings support previous studies that have also reported the co‐isolation of lipoprotein particles by SEC (Busatto et al., 2022; Sódar et al., 2016; Takov et al., 2017). This study utilised an apoB ELISA to quantify the presence of lipoprotein. ApoB was chosen as it is a main constituent protein of LDL, a known co‐isolate in EV preparations purified from serum. The higher amount of apoB found in MFC isolations may be a result of an overall greater particle and protein yield in the MFC isolations compared to SEC. When apoB is normalized to particle count there was no significant difference between MFC and SEC. This indicates that neither method was effective at depleting LDL and an additional method or isolation step would need to be introduced to efficiently remove lipoproteins. Overall, MFC and SEC yielded particles with similar purity from serum, however MFC presented as the superior choice due to achieving higher particle yields, and no significant difference in apoB content (Brennan et al., 2020; Tzaridis et al., 2021; Veerman et al., 2021).

On top of the aforementioned improvements in purity and yield of the MFC compared to SEC, MFC also has several practical benefits. MFC using the CC700 presents as an inexpensive and fast technique. The duration of EV purification using the CC700 is only around 15 min, and therefore it is far more time efficient than all other common techniques (Coumans et al., 2017; Doyle & Wang, 2019). Additionally, due to the flowthrough functionality of MFC, compared to SEC, much larger quantities of sample can be introduced to MFC columns without the need for extensive sample concentration, including quantities that exceed the column volume; EV purification is dependent on binding of contaminants to unsaturated resin as sample continuously flows through the column. With this in mind, MFC also presents greater scalability than SEC, as even when using larger columns SEC requires samples to be concentrated to ensure size exclusion effects are not hampered. Moreover, SEC resins operate within a fractionation range; anything outside of this range will not be separated. Custom made MFC columns or multiple MFC columns in series can increase binding capacity if required, although due to their high binding capacity, the size of column needed is likely to be far less than SEC. Additionally, MFC does not dilute samples to the same extent as SEC. This is particularly important when a small injection volume is used as it abolishes the need for further concentration after EV purification.

## 5 CONCLUSION

Overall, MFC yielded higher purity EVs than SEC from bioreactor preparations, and produced preparations with purities equivalent to SEC from 2D culture preparations and serum, but with significantly increased EV concentrations. However, while MFC produced increased yield and comparable purity of serum EVs compared to SEC, importantly co‐isolation from lipoprotein was not eliminated by either method highlighting the need for further development of EV purification techniques to remove these impurities. For large scale cultures, SEC must be performed multiple times on the same preparation or increased in size to achieve complete purification of EVs from bioreactor cultures, resulting in significant EV losses which defeats the purpose scaling up cell culture. MFC offers high scalability that SEC cannot. The findings reported in this manuscript indicate that MFC can be applied as a scalable and efficient alternative to SEC for EV purification.

## AUTHOR CONTRIBUTIONS

**Scott Bonner**: Conceptualization‐equal; data curation‐equal; formal analysis‐equal; investigation‐equal; methodology‐equal; validation‐equal; visualization‐equal; writing—original draft‐equal; writing—review and editing‐equal. **Simonides van de Wakker**: Conceptualization‐equal; data curation‐equal; formal analysis‐equal; investigation‐equal; methodology‐equal; validation‐equal; visualization‐equal; writing—original draft‐equal; writing—review and editing‐equal. **William Phillips**: Data curation‐equal; formal analysis‐equal; investigation‐equal; methodology‐equal; validation‐equal; visualization‐equal; writing—original draft‐equal; writing—review and editing‐equal. **Eduard Willms**: Conceptualization‐equal; supervision‐equal; writing—review and editing‐equal. **Joost P. G. Sluijter**: Resources‐equal; supervision‐equal; writing—review and editing‐equal. **Andrew Hill**: Resources‐equal; supervision‐equal; writing—review and editing‐equal. **Matthew Wood**: Resources‐equal; supervision‐equal; writing—review and editing‐equal. **Pieter Vader**: Conceptualization‐equal; supervision‐equal; writing—review and editing‐equal.

## CONFLICT OF INTEREST STATEMENT

PV serves on the scientific advisory board of Evox Therapeutics Ltd. SEB has equity and interests in Evox Therapeutics Ltd, and has held an employment contract with Evox Therapeutics Ltd., Oxford, UK, during the completion of this article. MJAW is a cofounder of Evox Therapeutics, Pepgen, Orfonyx and Isogenix. MJAW is a scientific advisory board member of Evox Therapeutics and Pepgen, and is a Director of Oxford University Innovation. The University of Oxford has patents issued and/or pending for oligonucleotide technologies and delivery technologies (on which MJAW is an inventor). Evox Therapeutics, Pepgen, Orfonyx and Isogenix had no influence on the conception or realisation of this article.

## Supporting information

Supplementary Figure S1. Supporting chromatograms from various applications to SEC and MFC columns. (A) 2 mL of DMEM + 1% antibiotics subjected to SEC using a Tricorn 10/300 column packed with Sepharose 4 Fast Flow resin. The resulting peak is close to identical to the “DMEM constituent peak” described in Figure 2a. (B) injection of penicillin/streptomycin subjected to a CC700 column. (C) Injection of supplement free MEM199 medium subjected to a CC700 column. (D) Direct injection of CM without proper buffer exchange in TFF.
Supplementary Figure S2. AIEX anion exchange following MFC purification presents an option to purify EVs from nucleic acids. (A) Nucleic acid content determined by Quant‐iT RiboGreen RNA Assay. (B) Particle yields determined by NTA. (C) Particle/µg of nucleic acids. (D) Mean particle size distributions determined by NTA (n = 3). (E) TEM images from the AIEX flow through and the elution phase. (F) Western blot analysis of EV proteins alix, β‐actin, syntenin, CD81, annexin A1 and TSG101. Equal protein amounts (10 µg) were loaded. Results in A, B, and C present n≥3 biological replicates.
Supplementary Figure S3. Complete western blots of human serum samples isolated by SEC or MFC supporting the blots shown in Figure 6H. Western blotting of (A) alix, (B) CD81, (C) flotillin, (D) TSG101 and (E) syntenin.

## Data Availability

The data that support the findings of this study are available on request from the corresponding author.
