# Supplementary material for: Scalable purification of extracellular vesicles with high yield and purity using multimodal flowthrough chromatography
Source: J Extracell Biol. 2024 Jan 31;3(2):e138. doi: 10.1002/jex2.138 (PMC11080796; doi:10.1002/jex2.138)
Supplement: Supplementary file 1 — Supplementary Figure S1. Supporting chromatograms from various applications to SEC and MFC columns. (A) 2 mL of DMEM + 1% antibiotics subjected to SEC using a Tricorn 10/300 column packed with Sepharose 4 Fast Flow resin. The resulting peak is close to identical to the “DMEM constituent peak” described in Figure 2a. (B) injection of penicillin/streptomycin subjected to a CC700 column. (C) Injection of supplement free MEM199 medium subjected to a CC700 column. (D) Direct injection of CM without proper buffer exchange in TFF. Supplementary Figure S2. AIEX anion exchange following MFC purification presents an option to purify EVs from nucleic acids. (A) Nucleic acid content determined by Quant‐iT RiboGreen RNA Assay. (B) Particle yields determined by NTA. (C) Particle/µg of nucleic acids. (D) Mean particle size distributions determined by NTA (n = 3). (E) TEM images from the AIEX flow through and the elution phase. (F) Western blot analysis of EV proteins alix, β‐actin, syntenin, CD81, annexin A1 and TSG101. Equal protein amounts (10 µg) were loaded. Results in A, B, and C present n≥3 biological replicates. Supplementary Figure S3. Complete western blots of human serum samples isolated by SEC or MFC supporting the blots shown in Figure 6H. Western blotting of (A) alix, (B) CD81, (C) flotillin, (D) TSG101 and (E) syntenin. [file JEX2-3-e138-s001.docx]

**Supplementary figures**


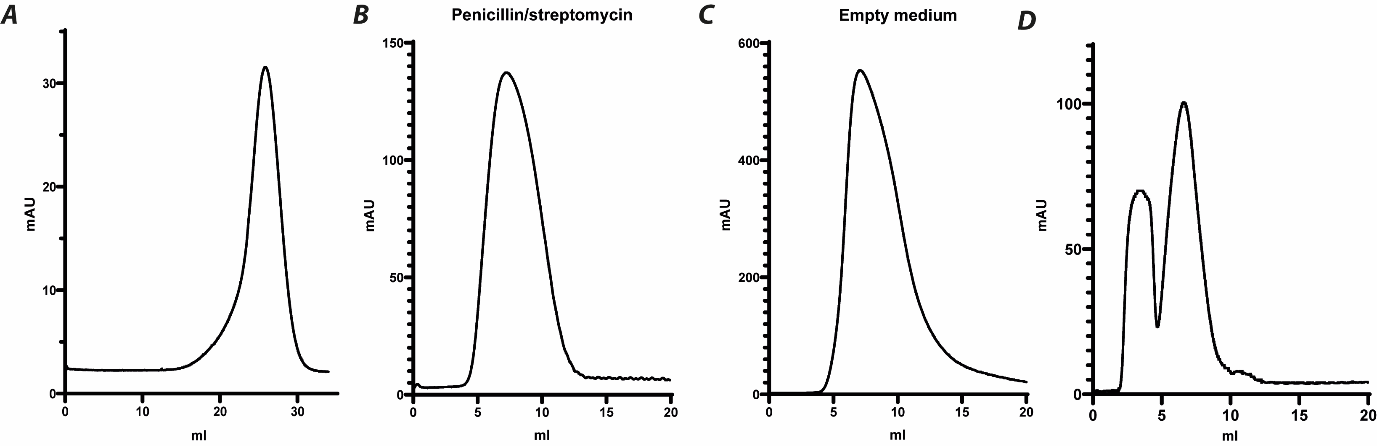


***Supplementary Figure S1****.* ***Supporting chromatograms from various applications to SEC and MFC columns.*** *(****A****) 2 ml of DMEM + 1% antibiotics subjected to SEC using a Tricorn 10/300 column packed with Sepharose 4 Fast Flow resin. The resulting peak is close to identical to the “DMEM constituent peak” described in Figure 2A. (****B****) injection of penicillin/streptomycin subjected to a CC700 column. (****C****) Injection of supplement free MEM199 medium subjected to a CC700 column. (****D****) Direct injection of CM without proper buffer exchange in TFF.*


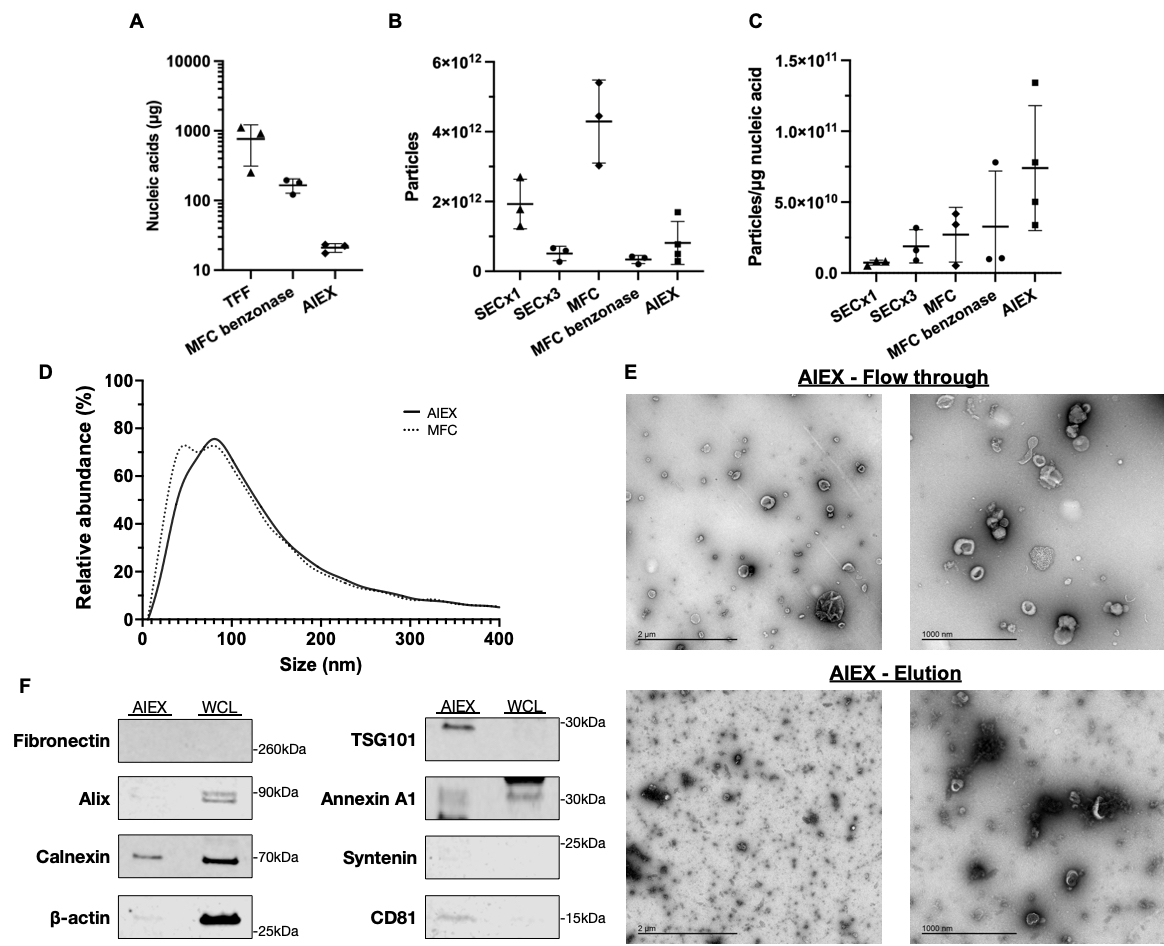


***Supplementary Figure S2.* *AIEX anion exchange following MFC purification presents an option to purify EVs from nucleic acids.*** *(****A****) Nucleic acid content determined by Quant-iT RiboGreen RNA Assay. (****B****) Particle yields determined by NTA. (****C****) Particle/µg of nucleic acids. (****D****) Mean particle size distributions determined by NTA (n=3). (****E****) TEM images from the AIEX flow through and the elution phase. (****F****) Western blot analysis of EV proteins alix, β-actin, syntenin, CD81, annexin A1 and TSG101. Equal protein amounts (10 µg) were loaded. Results in A, B, and C present n≥3 biological replicates.*


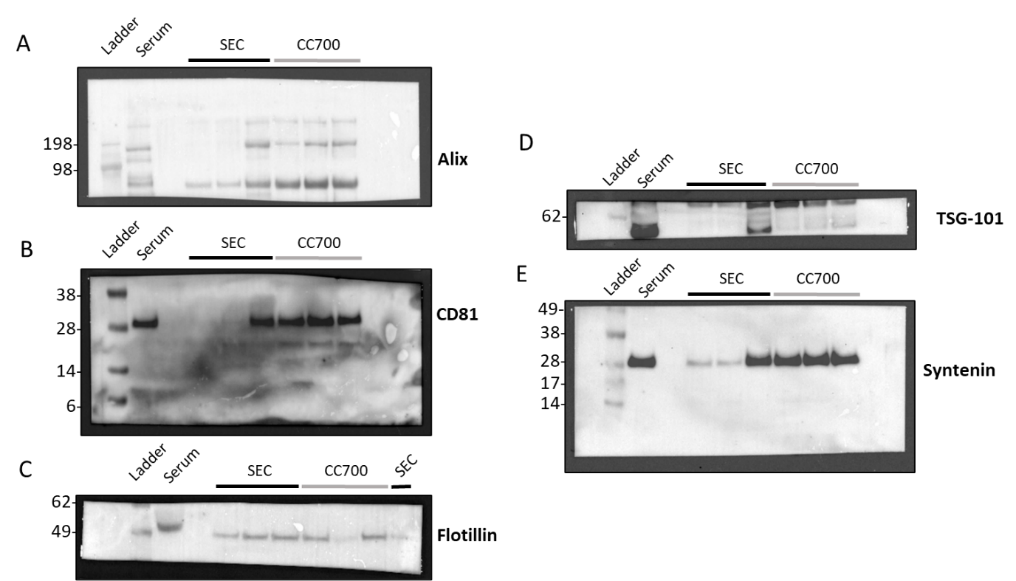


***Supplementary Figure S3. Complete western blots of human serum samples isolated by SEC or MFC supporting the blots shown in Figure 6H.*** *Western blotting of (****A****) alix, (****B****) CD81, (****C****) flotillin, (****D****) TSG101 and (****E****) syntenin.*
